# Comparison of McKeown Minimally Invasive Esophagectomy vs sweet esophagectomy for esophageal squamous cell carcinoma: A retrospective study

**DOI:** 10.3389/fonc.2022.1009315

**Published:** 2022-12-19

**Authors:** Fan Yu, Yaozhong Zhang, Haidi Xu, Kuankuan Li, Jingge Gheng, Chenxi Lin, Lei Li, Na Wang, Lei Wang

**Affiliations:** ^1^ Department of Thoracic Surgery, The Fourth Hospital of Hebei Medical University, Shijiazhuang, China; ^2^ Department of infectious disease, the Fourth Hospital of Hebei Medical University, Shijiazhuang, China; ^3^ Department of Molecular Biology, the Fourth Hospital of Hebei Medical University, Shijiazhuang, China

**Keywords:** esophageal squamous cell carcinoma (ESCC), neoadjuvant therapy, immunotherapy, McKeown minimally invasive esophagectomy, sweet esophagectomy

## Abstract

There are two most widely used transthoracic esophagectomy methods: the McKeown Minimally Invasive esophagectomy (McKeown MIE) and the Sweet Esophagectomy. We evaluated and compared the therapeutic effects of these two methods to determine the appropriate method for the treatment of middle and lower third esophageal cancer patients who received neoadjuvant chemotherapy combined with immunotherapy (NACI). We retrospectively analyzed 43 sweet esophagectomy cases received NACI and 167 cases with McKeown MIE in the fourth hospital of Hebei Medical University from December 2019 to May 2022. This retrospective observational study showed that Sweet esophagectomy and McKeown MIE after NACI therapy for resectable ESCC patients appeared to be safe with low operative mortality and morbidity rate in the current population. In addition, sweet esophagectomy was associated with a lower incidence of severe complications and shorter hospital stay for patients over 70 years of age compared with McKeown MIE. There were no differences were found in length of stay, mortality and complication incidence rate between the two groups. The Sweet approach has advantage in hospital stay for the treatment of the elderly NACI patients with middle or lower third esophageal squamous cell carcinoma. In conclusion, Sweet esophagectomy and McKeown MIE are both safe, effective, and worthwhile approaches for ESCC patients in immunotherapy age.

## Introduction

Esophageal cancer ranks the eighth most commonly diagnosed cancer and the sixth most common cause of cancer-related mortality worldwide (approximately 604,000 new cases and 544,000 deaths in 2020) (1); and the incidence of esophageal cancer ranked the sixth and the mortality ranked the fourth in China, based on the latest data from China’s national cancer center (2). There are two common pathological types of esophageal cancer: esophageal adenocarcinoma and esophageal squamous cell carcinoma (ESCC), and the histological type of about 90% esophageal cancer patients is ESCC (3). Many ESCC patients who received surgery alone have the poor treatment effect and prognosis. Neoadjuvant therapies have come into use to improve long-term survival rate (4–6). However, the 5-year overall survival rate was only 47%, and 3-year disease free survival was about 49% (6), even neoadjuvant therapies were applied. Therefore, it is important to establish novel and effective treatment strategies for ESCC to further improve survival.

The PD-(L)1 immune checkpoint inhibitors have showed encouraging treatment effect of patients with ESCC. Immunotherapy has been proved that, in the treat of unresectable advanced esophageal cancer, it has a good curative effect. Recent studies suggested that neoadjuvant immunotherapy could improve the prognosis of ESCC patients, and immunotherapy drugs have been proved to be effective and safe for tumors (7, 8). Such as “Camrelizumab, Durvalumab, Pembrolizumab, Tislelizumab, Toripalimab and Sintilimab” etc. (9–12), many kinds of immunotherapy drugs had been tested in clinical trials to evaluate the safety and efficacy in ESCC neoadjuvant therapy.

Up to now, the Sweet procedure is still widely used in China; and the McKeown MIE is being applied more and more often (13, 14). The Sweet esophagectomy and the McKeown MIE are two common surgical approaches for treating middle or lower ESCC patients. In most cases, a transthoracic esophagectomy with gastric tube reconstruction is performed cervical anastomosis (McKeown) (14, 15). We evaluated and compared the efficacy and feasibility of the Sweet esophagectomy and McKeown minimally invasive esophagectomy (MIE) following NACI to determine the better method for treating middle and lower third esophageal squamous cell carcinomas in immunotherapy age.

## Materials and methods

### Patients and methods

Our retrospective study was conducted at the Fourth Hospital of Hebei Medical University. Fourth Hospital of Hebei Medical University is an ultra-high-volume tertiary thoracic surgery center in north China, with about 1200 esophageal procedures performed in 2021. This research was approved by the Ethics Committee of Fourth Hospital of Hebei Medical University, China, and followed the principles of the Helsinki Declaration. All data were retrospectively collected on patients who underwent esophagectomy in the Fourth Hospital of Hebei Medical University between December 2019 and May 2022. Patients with histologic diagnosis of ESCC underwent neoadjuvant chemotherapy combined with immunotherapy (NACI) were included, and all included patients must be with available clinicopathological characteristics and personal information. We excluded the patients with preoperative superior mediastinum lymph node metastasis, higher lesion location (above the level of the carina), and benign tumors. Patients recruited in blind clinical trials, unavailable clinicopathological characteristics were also excluded from the selected samples. All patients received neoadjuvant chemotherapy, and the recommended regimens included paclitaxel (135–175 mg/m2 i.v, d1, q3w) plus cisplatin (75 mg/m2 i.v, d1, q3w) or nedaplatin (100 mg/m2 i.v, d1, q3w) or oxaliplatin (85 mg/m2 i.v, d1, q3w), etc. Five PD-(L)1 blockades that contained pembrolizumab (200 mg/kg, i.v, q3w), tislelizumab (200 mg, i.v, q3w), camrelizumab (200 mg, i.v, q3w), sintilimab (200 mg, i.v, q3w).

Simultaneously, a comprehensive pre-operative evaluation consisting of clinical presentation, physical examination, pulmonary function tests, electrocardiography, cardiac echocardiography, contrast-enhanced computed tomography (CT) scans of the chest and abdomen, and barium meal assessment, was applied to each patient to provide reliable information on the anatomy and anomalies for operative (16). All enrolled patients were without enlarged lymph nodes in the upper mediastinum (>5mm in diameter).

The pathological TNM stage was staged according to the 8th edition American Joint Committee on Cancer/Union for International Cancer Control staging system (17). We used CAP/NCCN(Ryan) system to classify regressive changes after neoadjuvant treatment based on histopathological results to reveal prognostic information (18, 19). Pathologic complete response (pCR), NCCN 0, was defined as no evidence of residual tumor cells of the complete resected tumor specimen of neoadjuvant therapy and resection. However, pCR patients might have regional lymph node metastasis (20). Operative time was obtained from the operating room nurse record, which was defined as the time from skin incision to closure. Surgical complications were evaluated and recorded according to the criteria defined by the Society of Thoracic Surgeons and the European Society of Thoracic Surgeons general thoracic surgery databases (21). R0 resection was defined as a microscopically margin-negative resection without microscopic tumor on the primary tumor bed. Disease-free survival (DFS) was defined as the time from the date of surgery to recurrence or death by any cause. All patients routinely came back to the hospital for a check every 3 months during the first 2 years after surgery, and then every 6 months after 2 years.

### Surgical technique

In the Sweet esophagectomy, the ESCC patients were placed in the right lateral decubitus position. A left posterolateral thoracotomy is performed through the fifth or sixth intercostal incision. The dissection of esophagus was performed through sharp and blunt procedure and was at least 5 cm above lesions. Careful intraoperative dissection was taken to avoid injury to the thoracic duct, the left vagus nerve, and the recurrent laryngeal nerve. The diaphragm was entered though a 5-to 6-cm radial incision, when the esophagus was completely freed. The left gastric artery and vein were ligated at their origins. The right gastroepiploic artery and arcades were carefully preserved. Meanwhile, a complete upper abdominal and distal mediastinal lymph node dissection was performed with an bloc resection of the distal esophagus and proximal stomach. Standard preparation of the stomach tube was performed in the left chest through diaphragmatic incision with a mechanical esophagogastric anastomosis above or below the aortic arch. In our study, no patient in the Sweet group underwent surgery through the combined thoracoabdominal approach, which increased postoperative pain for a costal cartilage incision (13).

In the McKeown MIE, the patient was placed in the left lateral decubitus position at first. Three ports were made: a 1 cm optical port was placed in the 7th intercostal space at mid-axillary line; the utility port, a 2 cm incision expanded with a protection sleeve, was placed in the 5th intercostal space at the anterior axillary line; the other port was 1.5 cm incisions placed in scapular line at the 8th intercostal space. The right recurrent laryngeal nerve lymph nodes were dissected and the mediastinal pleura were exposed at the level of the inferior pulmonary vein to dissect the esophagus. The azygos venous arcade was ligated to expose the esophagus. Meanwhile, an aggressive mediastinal regional lymphadenectomy was carried out. If necessary, the thoracic duct was also mobilized and ligated. After completing the thoracoscopic procedure, the patient was rotated to a dorsal decubitus, with the neck extended and turned toward the right. Pneumoperitoneum was established with 12−15 mmHg with CO2, following which, five abdominal trocars were inserted, a forcep is placed through the 5 mm trocar below the xiphoid process to grasp the gastrohepatic ligament for liver retraction. The mobilization of neck esophagus, left neck lymphadenectomy and construction of gastric conduit (diameter 3−5 cm) has been previously described (22, 23). With the control and skill of the technique, a lot of surgical refinements were added to the knowledge on technology. Ultimately, the gastric conduit was pulled up to the left neck through the posterior mediastinum (24).

All patients undergoing the Sweet Esophagectomy or the McKeown MIE had a nasogastric tube and a nasojejunal tube placed for postoperative feeding.

### Statistical analysis

Statistical analysis was performed after the completion of data collection and verification. Data were expressed as median and range unless otherwise indicated. Comparisons between different groups were evaluated using the Mann–Whitney U test, the Chi-square test or Fisher’s exact test. DFS was analyzed with the Kaplan–Meier method. All statistical testing is two-tailed and performed at the 5% significance level. Statistical calculations were conducted with SPSS software (IBM SPSS Statistics for Windows, version 26.0., IBM Corp., Armonk, NY, USA) and GraphPad Prism 9 (GraphPad, San Diego, CA, USA) (25).

## Results

### Baseline demographic and clinical characteristics

**Figure 1:**
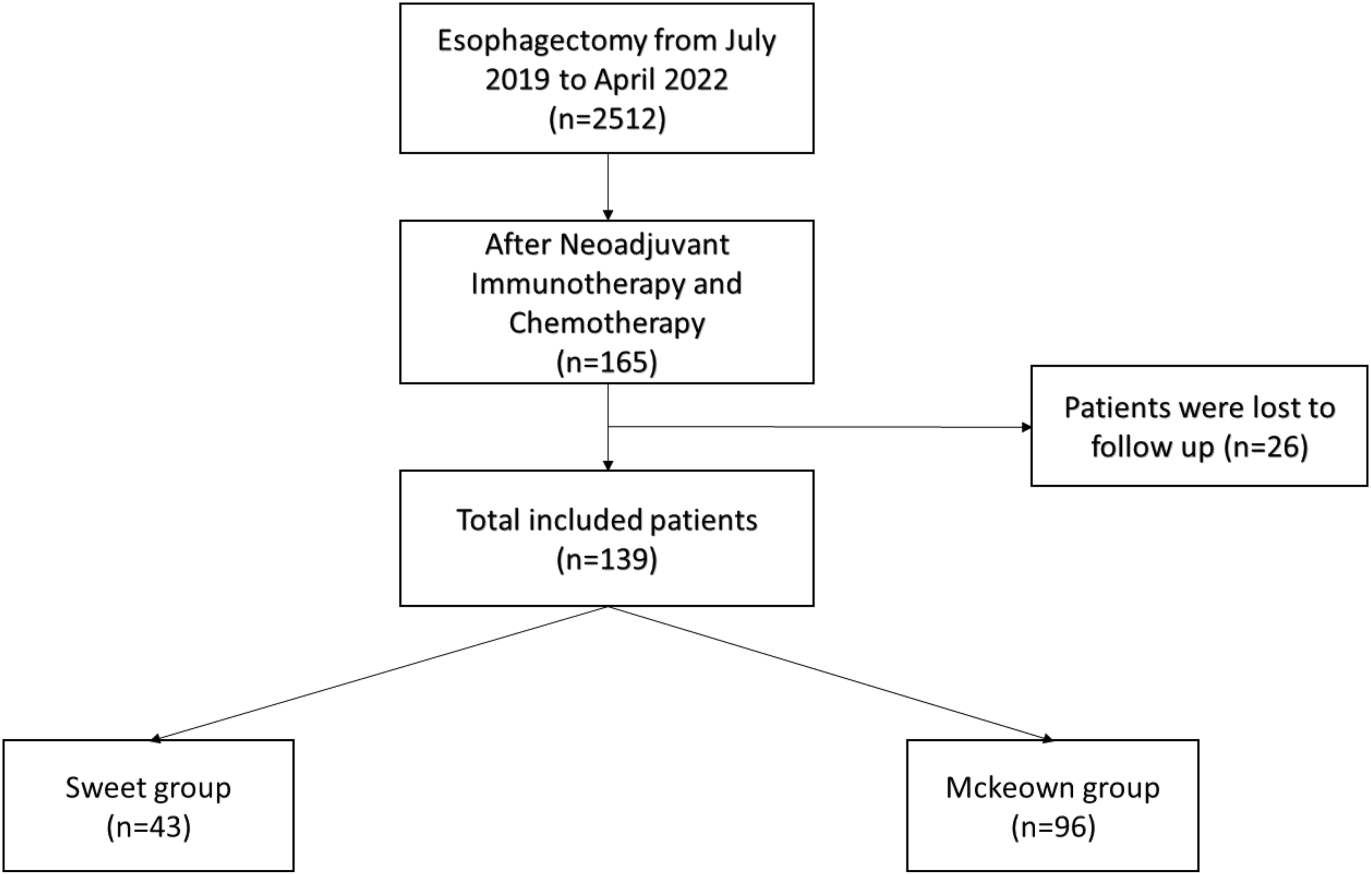
Flow diagram of the study population selection.

A total of 139 patients were enrolled in this study—43 patients (30.93%) in the Sweet group and 96 patients (69.07%) in the McKeown MIE group (**Figure 1**). The preoperative clinical data of patients assigned to the two groups are listed (**Table 1**). There were 102 males and 27 females in our study. Stage III ESCC, including IIIA and IIIB, accounted for 65% of these patients (57/139). Detailed postoperative pathologic reports were listed below (**Table 2**), and there was no significant difference found between two groups.

**Table 1:** Baseline demographics and clinical characteristics.

Characteristics	Sweet (n=43)	McKeown (n=96)	*P* Value
Age, year			
≥70	17	25	0.109
<70	26	71	
Sex			
Male	36	66	0.065
Female	7	30	
BMI, kg/m2			
Median	22.75	24.70	0.563
Range	18.25-30.46	16.00-31.43	
Tumor location			
Middle third	29	63	0.834
Distal third	14	33	
Clinical T stage			
cT1-2	14	43	0.324
cT3	19	31	
cT4	10	22	
Clinical N stage			
cN0	23	60	0.551
cN1	15	25	
cN2	5	11	
Clinical stage			
I	10	30	0.634
II	16	26	
IIIA	11	24	
IIIB	6	16	
Smoking history			
Never	22	46	0.819
Former	16	35	
Current	5	15	
Drinking history			
Never	22	48	0.382
Former	13	21	
Current	8	27	

**Table 2:** Histological Parameters.

	Groups, No. (%)		
Parameter	Sweet (n=43)	McKeown (n=96)	P Value
Tumor length, median (range)	3.0 (0.5-7.5)	2.5 (0.5-8.0)	0.365
Tumor Stage			
T0	9	37	0.126
T1	12	18	
T2	4	9	
T3	17	29	
T4a	1	3	
Nodal status			
N0	26	67	0.490
N1 (1–6)	14	19	
N2 (6–9)	2	4	
N3 (≥9)	1	6	
ypTNM stage			
I	19	54	0.407
II	8	11	
IIIA	9	13	
IIIB	5	10	
IV	2	8	

### Morbidity and mortality

Although operating time was significantly longer in the McKeown MIE group than in the Sweet group (mean [SD], 215 [36] minutes vs 174 [35] minutes, respectively; P <0.001); However, the hospital stay did not differ significantly between the two groups (median, 10 days in the Sweet group vs 11.5 days in the McKeown MIE group; P = 0.358). The overall incidence of patients having at least 1 postoperative complication was 35% in our trial. In the McKeown MIE group, only one patient (1.2%) underwent reoperation: all for control of chylothorax. And, no patient had reoperation in the Sweet group. There were no deaths reported (**Table 3**). For elderly patients (≥70), the hospital stay was longer in the McKeown MIE group (median, 10 days in the Sweet group vs 14 days in the McKeown MIE group; P = 0.014). The Sweet group also showed fewer serious complications in patients over 70 years old (**Table 4**).

**Table 3:** Postoperative Outcomes.

	Groups, No. (%)		
Outcome	Sweet(n=43)	McKeown(n=96)	P value
Intraoperative data			
Operative time, mean (SD), min	282 (38)	351 (36)	0.001
Blood transfusion	7	18	0.726
ICU stay, median (range), d	1 (0-12)	1 (0-14)	0.418
Hospital stay, median (range), d	10 (7-36)	11.5 (7-51)	0.358
Postoperative complications			
Anastomotic leakage	3	9	0.754
Anastomotic stenosis	2	7	0.721
Pulmonary infection	16	34	0.839
Cardiac complication	8	21	0.661
Chylothorax	0	1	0.691
Pleural effusion	12	30	0.692
Total	18	35	0.068
Incisal margin			
R0	40	92	0.287
R1	2	4	
R2	1	0	
Reoperations	0	1	0.691
In-hospital mortality	0	0	–

**Table 4:** Postoperative Outcomes in patients over 70 years old.

	Groups, No. (%)		
Outcome	Sweet(n=17)	McKeown(n=25)	P value
Hospital stay, median (range), d	10 (8-26)	14 (7-43)	0.014
Postoperative complications			
Anastomotic leakage	2	4	0.534
Anastomotic stenosis	0	5	0.062
Pulmonary infection	4	13	0.065
Cardiac complication	5	10	0.356
Chylothorax	0	0	–
Pleural effusion	5	13	0.109
Total	5	16	0.028

### Lymphadenectomy

**Figure 2:**
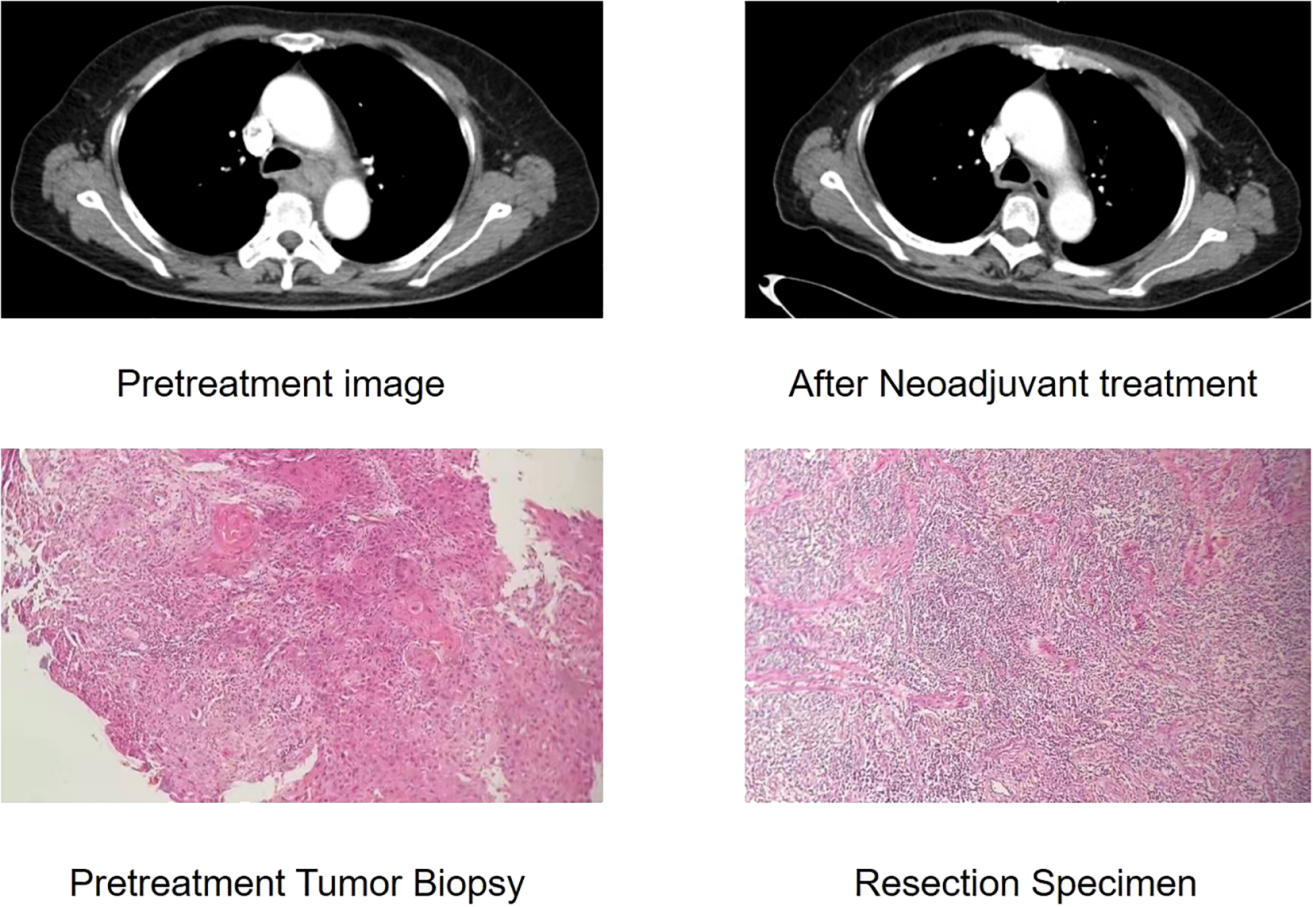
Case of radiological and pathological responses after neoadjuvant immunotherapy plus chemotherapy. This shows the radiological and pathological images of a 61-year-old male with a stage III(cTNM)ESCC before neoadjuvant treatment. The CT image shows insignificant shrinkage for the primary tumour. This patient achieved pathological regression of 95% for esophageal lesion with no residual lymph node metastasis according to postoperative specimen.

The McKeown MIE showed superiority in the dissection of lymph nodes in the upper mediastinum compared with the Sweet (median, 3 in the Sweet group vs 7 in the McKeown MIE group; P = 0.001). However, the number of lymph nodes retrieved in the middle/lower esophagus and perigastric regions was similar between the two groups (**Table 5**). 44 patients (44/139, 31.6%) achieved pCR in primary lesions, however, some had residual cancer cells in resected lymph nodes. One case of radiological and pathological responses were presented in **Figure 2**. We also estimated the metastasis rates of the lymph nodes in the 139 patients who underwent surgical resection (**Table 6**). For patients who achieved pCR (CAP/NCCN(Ryan)0), the metastasis rate of the upper mediastinum lymph nodes was only 7.6%, while it was 23.8% for the no evident tumor regression patients (CAP/NCCN(Ryan)3). Most notably, for patients who achieved pCR (CAP/NCCN(Ryan)0), the metastasis rates of upper mediastinum, middle mediastinum and perigastric were all between 5% and 10%; and no lymph nodes metastasis was found in lower mediastinum and celiac areas.

**Table 5:** Number of Lymph Nodes Resected.

	Groups, Median (Range)		
Region	Sweet (n=43)	McKeown (n=96)	P Value
Mediastinum			
Upper	3 (0–15)	7 (0-23)	0.001
Middle	6 (0-15)	4 (0-22)	0.070
Lower	3 (0-18)	3 (0-17)	0.563
Perigastric	10(0-29)	9 (2-27)	0.315
Celiac	0 (0-5)	0 (0-10)	0.084
Total	23 (8-55)	24.5 (8-63)	0.525

**Table 6:** Lymph Nodes Metastasis Rates of different regions After NACI.

	CAP/NCCN (Ryan)			
Region	0 (n=44)	1 (n=13)	2 (n=31)	3 (n=51)
Mediastinum				
Upper	7.6% (3/39)	27.2% (3/11)	23.1% (6/26)	23.8% (10/42)
Middle	6.9% (3/43)	8.3% (1/12)	6.6% (2/30)	27.6% (13/47)
Lower	–(0/42)	–(0/11)	16.1% (5/31)	18.7% (9/48)
Perigastric	6.8% (3/44)	38.4% (5/13)	23.3% (7/30)	35.3% (18/51)
Celiac	–(0/11)	–(0/4)	–(0/8)	–(0/13)

NACI, neoadjuvant chemotherapy combined with immunotherapy.

–, unable to calculate.

### Follow-up

**Figure 3:**
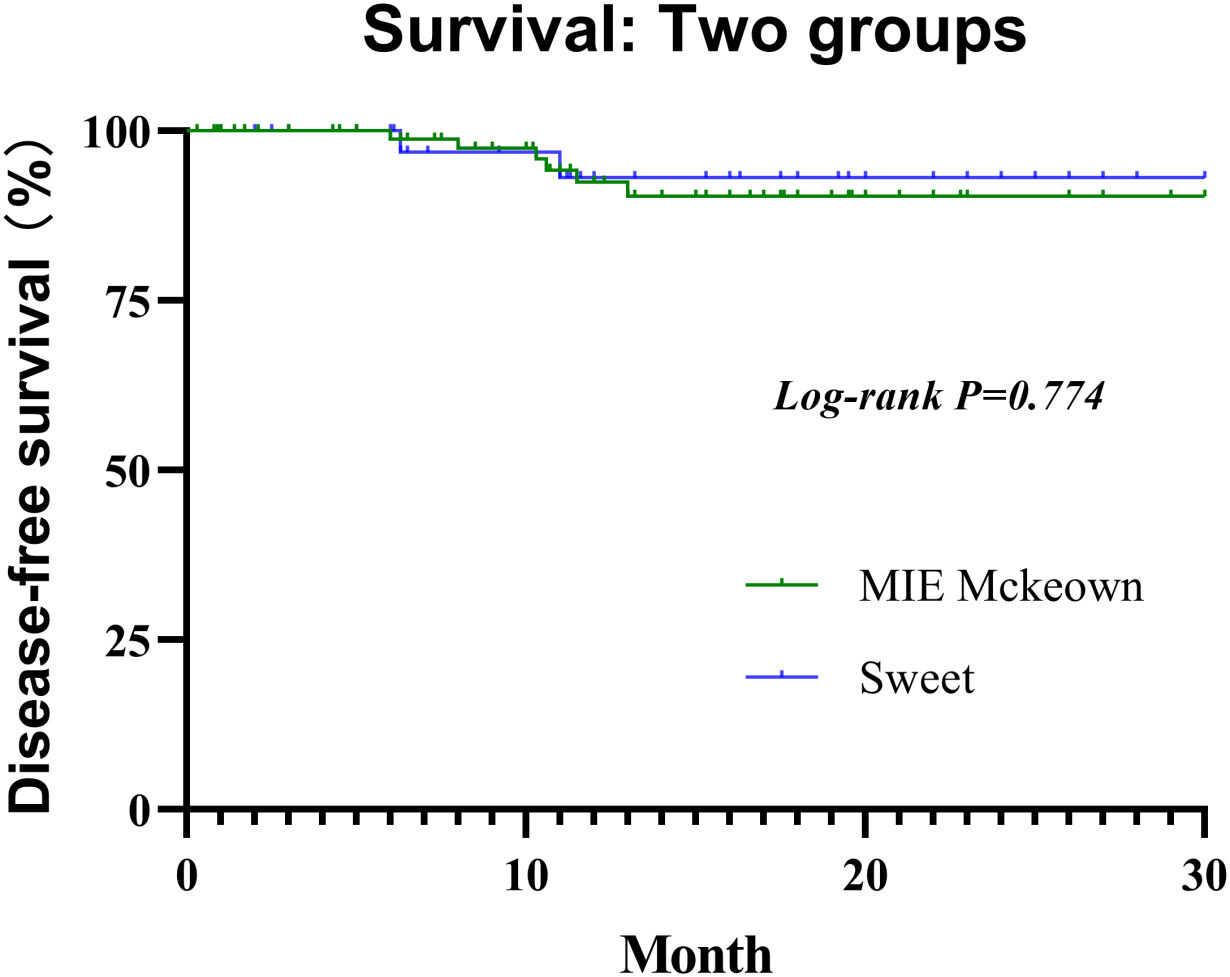
Kaplan–Meier estimates of DFS. DFS: Disease-free survival.

All selected patients were followed for 0.3 to 30 months (median, 11.6 months) until the cutoff day. he 1-year DFS rate of the Sweet group and the McKeown MIE group were 94.3% (95% CI, 85.3%–100%) and 95.2% (95% CI, 88.8%–100%), respectively (**Figure 3**). Not any median DFS was reached. In the Sweet group, one patient developed recurrence on the 6.3 months for the live metastasis, and the other patient was for lymph node metastasis on 11.0 months. Meanwhile, there were six patients in the McKeown MIE group developed recurrence;four for lymph node metastasis, one for live metastasis and one for pulmonary metastasis. The time of recurrence was 6, 8, 10.3, 10.6, 11.5 and 13 months, respectively. Although they achieved R0 resection, their pathological stages at baseline were IVA or III, and none achieve pCR. There was no significant difference between groups with regard to postoperative locoregional and distant recurrence, and no deaths reported.

## Discussion

This retrospective observational study showed that Sweet esophagectomy and McKeown MIE for resectable ESCC patients after NACI therapy, appeared to be safe. Both two operation methods showed low operative mortality and less complication than previous studies (26, 27). In addition, Sweet esophagectmy, compared with the McKeown MIE, was associated with a lower rate of severe complications and shorter hospitalization time in ESCC patients over 70 years old. There was no significant difference between the two groups in the length of stay in intensive care unit, operative mortality and postoperative complication rate.

Esophagectomy is still the most important treatment for patients with ESCC (28). However, mere surgery always is associated with high recurrence and metastasis rates (26). Neoadjuvant chemoradiotherapy (nCRT) plus surgery has become the mainstream treatment for the esophageal cancer patients in Western world to prolong life survival (27). However, neoadjuvant chemotherapy (nCT) without radiotherapy is advocated as a standard treatment for ESCC patients according to the JCOG9907 trial conducted in Japan (29). However, the adverse events and long-term survival of nCT plus esophagectomy or nCRT plus esophagectomy are still not satisfactory. The present studies and real-world data (20, 30–32), including PALACE-1, have revealed that neoadjuvant chemotherapy combined with immunotherapy (NACI) could provide encouraging pCR rate with good tolerability for resectable locally advanced ESCC (33). Patients who get MPR after neoadjuvant therapy are more likely to have better survival. Analyses of previous studies indicated surgery of after NACI therapy was safe and reliable (20, 34, 35). However, most NACI patients had vascular sheath thickening, surrounding tissues edema, increased capillary fragility and lymph nodes shrinking; and these might increase the difficulty of operation and the risk of intraoperative bleeding. Currently, no standard surgical procedure exists to the NACI patients.

To date, there are three main surgical approaches:McKeown, Ivor-Lewis and Sweet esophagectomy. In addition, the minimally invasive esophagectomy (MIE) in clinical was also widely carried out. All three methods can be chosen for patients with middle and lower ESCC. A major criticism of the Sweet esophagectomy, left thoracic esophagectomy, is that left thoracic approach always caused difficulty to performed adequate lymphadenectomy. Lymph nodes in the upper mediastinum and upper abdomen were difficult to removed in the Sweet esophagectomy, because the poor exposure in these regions (36, 37). The lymph nodes included those along bilateral recurrent nerves in the upper mediastinum and along the common hepatic and celiac arteries in the upper abdomen. All patients enrolled in the Sweet group were without enlarged lymph nodes in the upper mediastinum (>5mm in diameter) in our study. Compared with other esophageal cancer centers, which may be due to the more left-sided thoracic approaches were performed and the surgeons were familiar with the surgical method. It is undoubtedly that Minimally invasive esophagectomy has many advantages in lymph node dissection. The superiority of the right thoracic esophagectomy in radical lymph node resection is widely accepted, and our trial also showed significantly better upper mediastinum lymph node resection in the MIE Mckewon than in the Sweet esophagectomy. Meanwhile, the MIE Mckewon did not show superiority of celiac lymph node retrieval.

Although the studies of Chen et al. and Duan H et al. showed that the right thoracic approach was associated with higher DFS and OS in patients with ESCC compared with the left thoracic approach, especially in patients with lymph node involvement and/or r1-2 resection margin (10, 24). Notably, none of the patients in these studies received neoadjuvant therapy. In our study, the median follow-up time was 11.6 months, and DFS was similar in the two groups (the McKeown MIE group and the Sweet group). One reason may be the low incidence rate of low lymph node metastasis in pCR patients. Several studies advocated three-field lymph node dissection (38, 39). However, Chen et al. and Yin et al. found that there was no improvement in OS or DFS after esophagectomy with three-field lymphadenectomy over two-field lymphadenectomy, for patients with middle and lower thoracic esophageal cancer (40).

The total incidence of patients with at least one postoperative complication was 35% in our study. Although no significant differences were observed between the Sweet group and the McKeown MIE group, the total patients with postoperative complications, in two groups, were 18 vs 35(*p*=0.068). we found that the morbidity of Sweet group was lower than the McKeown MIE group, but there were no statistical significance. The smaller amount patients of the Sweet group was partly due to one patient got two or more complications. The main common complications of the two operative methods included anastomotic leakage, chylothorax and pulmonary infection. Anastomotic leakage is an important issue in various kinds of surgical complications, for it can be fatal and will decline the quality of the patients life (41). The rate of leakage in our series was common with recent studies using intrathoracic stapling technique. Prior evidence indicated that outcomes could be improved by avoiding very low-volume providers (42). Chylothorax was another major postoperative complication, and the only one reoperation, in this trial, was caused by it. In our center, thoracic duct ligation was routinely performed in two procedures. One study supported the preventive effect of thoracic duct ligation on chylothorax demonstrated. There were also no clinically meaningful difference between the two groups were identified, for pulmonary infection and other complications, including cardiovascular events, pleural effusion, and anastomotic stenosis,

We noted that McKeown MIE procedure was our preferred approach and more widely performed during the period of this study. Sweet esophagectomy was also one of our major surgical approaches, and our chief surgeons performed at least 50 Sweet esophagectomy one year. Hence the comparison is valid.

The study involved several limitations. First, although the surgery was performed independently by 4 senior surgeons in our center; the results need to be further confirmed in further multicenter trials with more surgeons participating. Second, our trail only evaluated short-term efficacy; long-term follow-up (OS and DFS) is necessary to evaluate the long-term clinical benefits of different surgery approaches for ESCC, after NACI therapy. Third, only two ESCC patients underwent NACI received MIE Ivor-Lewis. Sufficiently large sample is lacking to compare MIE Ivor-Lewis with McKeown MIE or Sweet procedure (43). In addition, we did not evaluate postoperative function status, so we could not evaluate the postoperative quality of life in detail (44).

## Conclusions

The Sweet approach has advantage in hospital stay for the treatment of the elderly NACI patients with middle or lower third esophageal carcinomas. Both Sweet esophagectomy and McKeown MIE are safe, effective, and worthwhile approaches in modern thoracic surgery.

## Data availability statement

The raw data supporting the conclusions of this article will be made available by the authors, without undue reservation.

## Ethics statement

The studies involving human participants were reviewed and approved by the Ethics Committee of The Fourth Hospital of Hebei Medical. Written informed consent for participation was not required for this study in accordance with the national legislation and the institutional requirements.

## Author contributions

LW and NW designed the study. FY and YZ collected the data. YZ and HX analyzed and interpreted the data. KL, CL, and LL carried out the clinical treatment and management of the patients. LW and NW prepared the final draft. All authors contributed to the article and approved the submitted version.
